# Animistic pragmatism and native ways of knowing: adaptive strategies for overcoming the struggle for food in the sub-Arctic

**DOI:** 10.3402/ijch.v72i0.21224

**Published:** 2013-08-05

**Authors:** Raymond Anthony

**Affiliations:** Department of Philosophy, University of Alaska Anchorage, Anchorage, AK, USA

**Keywords:** Iñupiat, pragmatism, environmental philosophy, sub-Arctic/Arctic cultures, narrative ethics

## Abstract

**Background:**

Subsistence norms are part of the “ecosophy” or ecological philosophy of Alaska Native Peoples in the sub-Arctic, such as the Inupiat of Seward Peninsula. This kind of animistic pragmatism is a special source of practical wisdom that spans over thousands of years and which has been instrumental in the Iñupiat's struggle to survive and thrive in harsh and evolving environments.

**Objective:**

I hope to show how narrative in relationship to the “ecosophy” of Alaska Native peoples can help to promote a more ecological orientation to address food insecurity in rural communities in Alaska. Alaska Native ecosophy recommends central values and virtues necessary to help address concerns in Alaska's rural communities.

**Design:**

Here, I will tease out the nature of this “ecosophy” in terms of animistic pragmatism and then show why this form of pragmatism can be instrumental for problematizing multi-scalar, intergenerational, uncertain and complex environmental challenges like food security.

**Results:**

Native elders have been the embodiment of trans-generational distributed cognition,^1^ for example, collective memory, norms, information, knowledge, technical skills and experimental adaptive strategies. They are human “supercomputers,” historical epistemologists and moral philosophers of a sort who use narrative, a form of moral testimony, to help their communities face challenges and seize opportunities in the wake of an ever-changing landscape.

**Conclusions:**

The “ecosophy” of the Iñupiat of Seward Peninsula offers examples of “focal practices”, which are essential for environmental education. These focal practices instil key virtues, namely humility, gratitude, self-reliance, attentiveness, responsibility and responsiveness, that are necessary for subsistence living.

Come forth into the light of things, Let Nature be your teacher.

– William Wordsworth

*Food security* is often defined as “a situation that exists when all people, at all times, have physical, social and economic access to sufficient, safe and nutritious food that meets their dietary needs and food preferences for an active and healthy life” (1). Its component parts include availability, accessibility, acceptability, adequacy and/or agency. According to this metric, many indigenous Alaskans and those living in rural communities around the state are not food secure. This is both a practical concern and an ethical one.

Alaska Natives and rural villagers feel that they are in peril. Climate, political and economic stressors (e.g. a lack of equity in terms of allocation/distribution of resources, and anaemic political control and self-determination at local–tribal levels) are impediments to attaining subsistence resources for calories and nutrition, especially when access to and availability of traditional food sources and quality foods are inhibited. Furthermore, these stressors pose urgent challenges to traditional spiritual and practical lifeblood practices of Alaska Native communities, that is, to their very existence and culture. According to Elaine Abraham,^2^ a Tlingit elder, *northern* knowledge is becoming less and less relevant due to competing knowledge sources (often Western) that identifies self-worth with productive function and which rely on technological fixes that tend to focus on domination of the natural world. Native communities throughout the state ponder this urgent question posed by Abraham: “How do we teach our young people [about] the sacredness of our lands [and oceans]?”. Furthermore, as a result of the impacts of climate change in rural Alaska (e.g. Unalakleet and Shismaref), dwindling job opportunities in rural areas around the state and gradual fragmentation of traditional social structures, larger cities like Anchorage and Fairbanks have seen significant urban migration. New “immigrants” (many of whom do not have sufficient employment skills or education) to urban centres like Anchorage are unable to participate in shaping regulations and infrastructure that affect their present lives and futures. Alaska Native peoples will likely have to modify their way of life in significant ways in order to respond to these challenges.

Loss of traditional food sources, for example, the disappearance of certain staple species like seal, walrus and salmon, and changes in wildlife habitat and migratory patterns (2) result in significant hardships to communities living in coastal areas. There is a spike in illnesses such as diabetes, heart disease and obesity. Dental decay is also a pressing matter, especially among the youth as more and more Northern, for example, sub-Arctic villages in Alaska, are forced to rely on non-traditional food sources and highly processed foods.

## Objectives

In the pages that follow, I hope to show how narrative in relationship to the “ecosophy” of Alaska Native peoples can help to promote a more ecological orientation to address food insecurity in rural communities in Alaska. Alaska Native ecosophy recommends central values and virtues necessary to help address concerns in Alaska's rural communities. Focusing on the Iñupiat of Seward Peninsula,^3^ I argue that the subsistence constructs of these Alaska Native peoples, namely, a form of animistic pragmatism, are important not only for these members of Alaska's north, but they can also be instructive for environmental philosophers and policy-makers concerned with equity and promoting holistic and longer range solutions concerning the struggle to bring greater food security to remote and environmentally challenged places like Alaska's sub-Arctic. This essay also encourages greater attention to the “distributed cognition” of native elders as a way to learn how to live by central subsistence virtues and activities.

## Design: philosophical discussion

### Alaska Native ecosophy

Much of contemporary environmental philosophy is hierarchical and dichotomous in nature. The dominant view is that the “non-human” world has no moral significance in itself. It is the “Other” that we exploit because it has but instrumental value. Traditional human-centric ethics does not tend to locate the human being as a part of a large whole. We tend to create policies that reflect human and not ecological time and there is a push for control and domination of this “Other.” In contrast, Alaska Native “ecosophy” or ecological philosophy (a term attributed to Kawagley (3), a northern knowledge elder and scholar), attempts to soften the distinction between the human and non-human worlds. Native ecosophy stresses the role of nature, soil, sea, animals and climate in the formation of moral character (arguably, both private and public traits and dispositions, i.e. virtues). It has for thousands of years given Alaska Native Peoples a perspective on *Being* that focuses on the relatedness and interdependency of life forms (individuals and groups) within our mixed communities of humans, animals and plants. More importantly, it promotes a kind of moral ecology that encourages connectedness and connectivity to time, place, people and other beings. It can be seen as an environmental ethic that attempts to overcome estrangement with the “Other” by encouraging both integration and solidarity with nature and its constituents. Community members develop a certain level of competence (necessary skills to survive and flourish) and comprehension (knowledge of how things fit together and of how a healthy balance may be promoted). The non-human world is not treated as an amorphous group, and solutions to place-based challenges are not reductionistic as we may find in many contemporary Western responses to environmental concerns (which typically seeks a one-size-fits-all tech-fix to our environmental challenges). As an ethic of engagement, Native ecosophy (more broadly) shies away from exploitative hierarchies of consumerism and does not portray solutions in terms of mitigating human parasitism on ecosystems. Native ecosophy focuses on our connectedness to all other living things and on the importance of thinking about activities and policies in (longer range) ecological time and not just in human time (e.g. election cycles or 1 or 2 generations) when it comes to the fundamental mind-set to adapt to our changing circumstance.

### Narrative as normative moral testimony

Indigenous moral knowledge and knowledge transfer can underscore the importance of narrative in our moral deliberations. While steeped in history, narrative (as mirrored in the reservoir of collective wisdom) can encourage moral experimentation in response to an ever-changing world. The stories of the elders are based on the best evidence and moral reasons they have presently. With 1 foot in many yesterdays and another stretched to tomorrow the wisdom that encompasses thousands of years that we find in indigenous cultures like the Iñupiat can stretch our moral imaginations, especially in terms of finding solutions to environmental and food crises.

Narrative as an ethical medium encourages us to focus on building our human character structure to incorporate the virtues that will lead us to value and preserve what we have identified as important (morally and otherwise) for our own sake and others. As reflected here by storyteller, William Kittredge (4), elders also recognize that:

We live in stories. What we are is stories. We do things because of what is called character, and our character is formed by the stories we learn to live in. Late in the night we listen to our own breathing in the dark and rework our stories. We do it again the next morning, and all day long, before the looking glass of ourselves, reinventing reasons for our lives. Other than such storytelling there is no reason to things.

Narrative, as reflected in the testimonies of elders, play a substantive role in ethics by offering rich biographies and facts, and by drawing audiences to observe carefully and investigate comprehensively both present states of affairs and precedent histories. Narrative can be employed to motivate moral examination, explanation and justification. Narrative also encourages deeper (and different from analytic cogitations) philosophical inquiry regarding the constitutive structures of life itself. Native elders use narratives to pique the curiosity of listeners about the lives of the agents concerned. Narratives can occasion a certain kind of “living as if” one is the agent in the midst of a moral dilemma and “living through” her predicament. Narratives stir us to “see with” or “be as” the agents or subjects portrayed. Our moral and conceptual imaginations and capacity for sympathy and empathy are excited to appreciate the practical realities of the everyday life experiences that the agents or subjects portrayed in these narratives endure. Narratives invite audiences to apprehend what is morally relevant for subsequent attitude (re)examination and action (5, p. 44). Narratives express normativity through (6, p. 46):

- Content, that is, the substance out of which the narrative is constructed, which includes the agents and subjects portrayed, state of affairs and the concatenation of events;
- Organization of plot or point of view, that is, the story's “thematic unity” or scientific/empirical inferences. This includes the story's “teleological logic,” its unfolding and the position from which it is told; and
- “Performative conditions” under which the narrative is expressed and received. That is, how the alignment of the characters portrayed in the story make an ethical appeal to the audience as a way to facilitate identification with the roles, responsibilities, dispositions, attitudes and actions of the ensemble of main characters on the story. Elders may use the pathetic and ethical proofs of the narrative to engender sympathy, repulsion and other moral feelings, sentiments and dispositions as a way to evoke questions and reflection in the hearers’ about their own moral agency.

### The elder as historical epistemologists and moral philosopher: Iñupiat as animistic pragmatists

Alaska Native peoples like the Iñupiat are guided by their worldview to employ appropriate ecological knowledge, suitable practices and technologies in a prudent way as a means to keep exploitation of others and resources in check. The norms of sustainability are not based on grand theoretical systems of ethics. Morality is what is most practicable. Norms are drawn from the lived, shared experiences of ancestors and preceding elders. There is a moral–philosophical dimension that can teach us in the West how to relieve some of the tensions over the human-nature distinction. Some central subsistence values include balance, harmony, and reciprocity cum gratitude, respect for ecological time and not just the human time scale. These subsistence constructs are grounded knowledge or embodied truth in the wisdom of the elders. They are holistic, inductive, cumulative and instinctual and are recommended through “focal Practices” [coined by Borgmann, in Thompson (7)]. According to Borgmann, focal practices are habits or activities that “gather” us to participate in a continuing interconnectedness (reciprocal engagement) between humans and the natural world. Native narratives and practices encourage contact with the sacred, spiritual and virtuous habits. They promote a kind of moral ecology of virtue and apprehension that “[a]ll of life is considered recyclable and therefore requires certain ways of caring in order to maintain the cycle” (3, p. 10; see also ref. 8).

Native subsistence constructs are embodied in the person of elders, that is, persons who possess a particular kind of expertise. A “respected elder” as expert, is someone who offers consistent examples, and who reflects by doing the normative truth.^4^ She or he is:

- An historical epistemologist (which presumes deep or significant historical literacy of the culture), and
- A moral reflector (one who is a moral philosopher of a kind).

#### Historical literacy and historical epistemologist

To be an historical epistemologist, elders must be reservoirs of cultural narrative. To employ Wohlforth's term, they must be like “supercomputers” and have access to the cognitive prowess of generations/millennia (2). Native hunters, for example, are able to take massive amounts of data and process it quickly and effectively for a desirable outcome. The slide from historian to epistemologist with a moral flare involves the *process of reflection* on the stockpile of data (cognition/expertise that is dispersed/distributed, so to speak), much of which will include moral reasons that shape the narratives or is revealed in them. Elders reflect a lot on natural phenomena, for example, like relationships between human beings and environmental processes and they are valued for their observations and reflections. They are also valued for their ability to apply inherited wisdom that is responsive and apt to a novel situation. That is, the valued reflection is the enacted reflection or ancient wisdom in motion to address a modern moment.^5^

As pragmatists of a sort, native elders employ their enacted reflection to *frame* and promote a *process of inquiry* not dissimilar to the method proposed by pragmatists like (9, 10). The *mode of intercourse with a problem* (i.e. in the wake of “indeterminacy”) is *experimental* in nature. Elders may put forward hypotheses for testing with no pressure to have final solutions, only momentary and “at-the-time-better hypotheses” to help the community reflect on their current habits and their trajectories and to cope with challenges (both distal and proximate). As pragmatists of a sort, elders recognize that solutions are unfinished and dynamic, where moral dilemmas have many moving parts. That is, when one aspect or element of a moral dilemma may be resolved, other related ones may need to be addressed as a result. Elders may challenge hearers to participate in a process of *meaning clarification*, where core values and aims and central background conditions that underpin their habits are raised, reflected upon and scrutinized. Perceived and actual drivers (e.g. epistemological and ethical) that influence virtuous habits and which may corrupt individuals and social structures are identified along with risks that may jeopardize the community's coherence as such. The mode of intercourse with an indeterminate situation culminates in ethical assessment where interventions and strategies are tested to see if they produce satisfactory solutions. By applying ancient wisdom to their evolving circumstances, elders can help community members reflect on the nature of the interplay between the drivers that motivate their solutions and identify and potentially attempt to reconcile conflicting values and priorities. In this sense then, elders, arguably, are moral philosophers of the pragmatic persuasion.

Many elders embody or know the narratives with the intimacy of personal discovery. This intimacy gives them a particular understanding of the moral reasons behind specific normative truths conveyed through these stories. The inherited collective information (traditional or local knowledge) and the acquired verifications and insights into normative truths allow elders to apply moral reasons to evolving circumstances today.

#### Moral philosophers and forensic capability

To be a moral philosopher, elders embody a particular kind of forensic skill, namely storytelling or narrative. By reflecting tradition through behavior, moral elders serve as moral compasses for the community. On the one hand, as historical epistemologist, elders have gained “knowledge of World or Being”. She or he uses this knowledge as the basis for knowledge how and knowledge that. Furthermore, as moral philosopher, the elders can accurately and insightfully reinterpret ancient knowledge of being into knowledge that can be used in the modern moment. Here, an elder as expert is able to masterfully summon narrative to express normative belief and practice. Through narratives native elders not only convey moral knowledge but the narratives themselves, in some way, “authenticate” the storyteller as an elder/moral expert.

As moral philosopher, a “respected elder” can employ narrative to persuade by “presenting a logical explanation, a pathetic proof, and a palpable demonstration of good ethos” (6, p. 49, on narratives in general). By Liszka's lights, the *logical proof* exemplifies how the events in the narrative unfold in light of the plot or theme that is being developed and thus is connected to how well the thematic unity is developed and sustained. The *pathetic proof* refers to the kinds of emotions or cogitations the narrative evokes, that is, in what ways has the story “moved” or persuaded us. And lastly, the *ethos* of a narrative deals with the moral characters of the agents or subjects portrayed and the extent to which audiences are able to identify with them. Some may be led to personal transformations or to revisit their own moral underpinnings as a result.

Elders recognize that narrative is a vehicle for understanding moral truth.^6^ As moral philosophers, elders may use narratives as cultural anchors as much as cultural identifiers. Elders use narratives to help recipients explore the proclivities and motivations of the agents or subjects portrayed. They use narratives to express a point of view, through a constructed web of logical and/or emotive connections, and to challenge audiences to evaluate the predicaments of the agents involved from the moral point of view by examining and justifying the normative claims embedded within them (11). While narrative is not an “all-purpose solvent in which [pressing] issues of any kind at all could be efficiently disentangled, any and all conclusions neatly disengaged” (5, p. 19), it can enable recipients to “[see] a complex, concrete reality in a highly lucid and richly responsive way, [and to help take] in what is there, with imagination and feeling.” Thus, narrative, as a form of moral forensic (as Iñupiaq elders seem to have found), can promulgate significant moral insights and open new vistas of moral understanding and knowledge (5, p. 152).

In summary, for the Iñupiat (and many northern communities), the possibility of normative truth depends on reaching backwards to the past in order to leap forward. Elders are historical epistemologists and stewards of the instrument of normative knowledge. They rely on both collective memory and their powers of reflection to help thrust the present generation into the future in the wake of different challenges.

#### Subsistence constructs and food security

Many Alaska Native narratives focus on the profound and immediate connection with the environment. Iñupiaq elders help their audiences consider everything in their surroundings, for example, how they are connected to the agencies of animals like whale or salmon. The elders’ inherited wisdom and experiences help community members know just how to relate to animals meant for harvest. In the stories, unless the virtue of humility is embodied and expressed and respect for the salmon or whale's own agency observed, these animals will not come back. According to Iñupiaq ecosophy, the human epistemological relationship with subsistence species (management of wildlife so to speak) cannot be devoid of spirituality, which in turn relies on how we communicate or get information from nature and its constituents. Human hubris tends to lead to an outcomes based orientation that focuses on maximizing benefits (or minimizing harms) and away from listening to the needs of others in the system and human responsibilities to be good stewards. Iñupiat ecosophy focuses on processes that promote integration of others’ importance in the chain of being co-citizens of that system and not antagonistic competitors. Thus, Iñupiaq subsistence constructs are not teased out merely in economic or utilitarian terms but has important spiritual, cultural and experiential dimensions.

In terms of food security, subsistence constructs reinforce a view of sustainability as “functional integrity” (7). Under this view, subsistence concentrates on the reproducibility, over time, of the whole system. Subsistence a la sustainability is NOT about consumption or merely stretching resources over a period of time. Instead, the mind-set regarding sustainability is about harmonizing human interaction with nature. Every member of the environment is considered to be part of the living community and plays a part in contributing to place health and balance. All creatures are born equal and each “agency” (or spirit) fills a function or niche and must perform it well in order to contribute to place health and balance.^7^ Alaska Native ecosophy has much to offer thinkers in the academy who are mainly concerned with problem solving and who appreciate local contexts and adaptive strategies. The knowledge passed down through narratives in the persons of the elders is naturalistic, developmental, experimental, fallible and anti-speciest,^8^ bearing the hallmarks of a pragmatic normative approach to problem solving. Through narratives and focal practices that are embedded in indigenous traditions, subsistence cultures like the Iñupiat have a way to orient themselves (i.e. frame important questions regarding their position in nature), have the opportunity to consider hypotheses to resolve problems within the context of community discourse, deliberate in a way that serves both to clarifies and refine solutions, and the opportunity to test and evaluate their solutions to help establish parameters that reflect good stewardship of “resources” with respect for the different natural constituents with whom partnerships must be forged. The focus on Alaska Native ecosophy is on the idea that “knowledge without wisdom is dangerous.” Elders tend to impress this idea and the need to appreciate different kinds of epistemologies in their narratives. We would do well in Western academies to include among “the best available science,” Alaska Native ways of knowing.

In contrast to much of conventional management philosophy that tends to emphasize trade-offs between the “human world” and “non-human natural world”, in panenthistic animistic worldviews that are characteristic of Alaska Native people's cultures these 2 “worlds” are one, and therefore everything is natural and a part of nature, including humanity; dualistic division reflects a *very* Western cultural orientation. Alaska Native ecosophy as reflected in the narratives and wisdom of the elders provides guidance for food security cum environmental policy by addressing the needs of entire ecosystems since it recognizes the interdependent relationship of human beings to their natural environments. Human beings are contributors or partners to the structure and sustainability of ecosystems through their harvesting and conservation activities. The 2 main principles that have helped the Iñupiat and other northern subsistence cultures (for example) survive and thrive against difficult challenges include respect for interdependence and equity through cooperation. Subsistence constructs can offer management concepts for policy makers concerned about food security by focusing on a system of harvest (or production) that emphasizes the interdependence of life, by listening to the rhythms of living organisms and paying attention to ingredients that promote prosperity of places where we live instead of trying to dominate the natural world through technological fixes. The need for cooperation with all citizens in our “mixed communities” (12) is also a way in which we express the virtue of gratitude to life-sustaining partnerships. The mind-set of interdependent mutual respect can promote a culture of care about community and environment and may forestall disenfranchisement of people and other constituents in the food chain. The organic and cooperative attitude is based on nurturing the environment and community and may lead to empowerment of rural villages to take control of their own futures by harnessing traditional place-based knowledge. Emphasis on virtues of humility, reverence and gratitude towards nature is central for future generations to thrive on the land and by the sea.

## Results and conclusions

How may we promote a more pragmatic ecological orientation to address food insecurity concerns in places with similar or analogous circumstances like the one discussed here? We should continue to engage indigenous beliefs in and of itself apart from our academic commitment to diversity so that we may overcome the functional dismissal of indigenous wisdom. As discussed above, a brief but closer look at the Iñupiaq world (and other northern subsistence cultures for that matter) can give us insight into some interesting moral–philosophical questions and ways of framing and solving problems. We see the requirements of a moral expert in the Iñupiaq world (i.e. historical epistemologist and philosopher of sorts, employing the forensic skills of story-telling and narrative to convey normative truth and address problems with practical solutions in view). The Iñupiaq world illustrates the value of narrative as moral testimony, for example, in the Iñupiaq insistence on the agency of animals. This is clearly not mere anthropomorphism or a “treat as if” they are (to use the nomenclature of the Academy), “autonomous subjects.” Instead, the strong belief that the animal makes moral determination is an accepted normative truth. The Iñupiaq relationship with animals offers us a different paradigm that can help us in the West to transform the ways in which we think of animals, the flora, oceans and atmosphere and to see ourselves as working together with the different agents in our ecosystem to continue to promote its health and viability. This different ways of knowing/ecosophy can help in the development of solutions by suggesting the need for personal and collective action and different public institutions than currently exists perhaps to respond to climate change and food insecurity with a broader spatial–temporal view.

The Iñupiat of Seward Peninsula offer us a different kind of consciousness that is instructive about how we should relate to the non-human world and confront practical cum normative problems. Native elders, through stories can help us appreciate how the different facets of the ecosystem hang together and in effect, the range of duties that must be assumed by the different levels of human institutions in order to be food secure in harsh environments. The narratives that are distributed through Iñupiaq ecosophy (and thus over a period of ecological time) can encourage all of us to expand our consciousness and not to see Nature and its constituents as merely a productive, resource extraction system or as commodities. Narrative when used as a vehicle to persuade us to behave in certain ways can give us assurances that our commitments are the right ones to hold and it can also provide us with justification for our actions (19). Further, being resilient and living sustainably means recognizing the “functional integrity” of the places where we live. It means putting into place adaptive strategies and policies in a time of significant environmental challenges that are limber and contextualized, and which respect the circle and cycle of life that reaches back in time and beyond just the immediate frame. It also entails appreciating relationships at all levels of human–natural world interactions and *listening* to a diversity of narratives and voices.

## Footnotes

2 Elder Abraham shared her concerns on how the climate is impacting food security and the lives of Alaska Native peoples and their cultures at a meeting on Climate Scientists, Native Elders, Religious Leaders on Climate Change, Center for Advancing Faculty Excellence (CAFÉ) UAA/APU Difficult Dialogues and the UAA Sustainability Office, November, 2011.

3 Who are the Iñupiat? Briefly, the Inupiat are a northwest Alaska Native group who do not only live on the Seward Peninsula since the group extends as far south around the Norton Sound to Unalakleet. They live on the Peninsula above Kotzebue Sound that contains the city of Kotzebue, Point Hope and Barrow. The group extends along the northwest coast, and the north coast until it runs into an Inuit group near the border of Canada. Iñupiat may also be identified as Inuit and are related to Inuvialuit of Yukon and North West Territories.

4 The emphasis on “doing” here echoes Aristotle's view of those who exemplify moral excellences on his virtue ethics account.

5 I am indebted to Kristin Helweg Hanson for this insight among many others.

6 Narratives can also be used to mislead. Thus, it is important that informants establish their trustworthiness. Note, since much of Native knowledge is experimental knowledge when it comes to the environment, time-tested pragmatic success can add to one's credibility.

7 The notion that Alaska Native societies are stewards of the animals that they consume for food and commerce is inextricably intertwined with their cultural, economic and social identities. Subsistence hunting activities is a lifestyle and these activities are carefully timed and carried out in a manner that ensures sustainability and dignity of the animals. The Iñupiat believe that there is a finite number of animals that exist between the real and spirit worlds. In the confluence of human–animal interlocution, an animal that has allowed a hunter to kill it, as the narrative above indicates, is thought to have knowingly sacrificed itself for the benefit of the human being, “given” itself to the hunter, someone who is open to communicating and listening to the animal's spirit directly (13). If present hunts are respectful, then future animals will make themselves available to Iñupiat hunters in the future. It is believed that poorly treated animals remain in spirit form, and do not return to the world. From a practical standpoint, ill treatment of animals would result in fewer animals available for food (14,15). Hunters do not think of themselves as “killing” animals. Instead, animals are “invited” to participant in a reciprocal interdependency that aims at sustaining the ecological life of the constituents of a particular place for generations (13,15). Special observances relating to animals are integrated into everyday life activities as a constant reminder of maintaining a respectful attitude towards human–animal interdependencies. Respect for this “ministry of nature” [a phrase Wohlforth (2) attributes to Brower Sr.] is crucial to survival and flourishing in this harsh Alaskan landscape. Traditional Iñupiat believe that animals have spiritual agency [(14,16), currently unpublished dissertation; see also Linderman (17) who discusses the antelope in similar light]. This agency is reiterated and celebrated in the feast after the hunt so that the humans may be beneficiaries again. The feast celebrates communal solidarity and the animal, as agent, is an integral part of this community. When the animal as agent is regarded and celebrated as being in solidarity with the community, the distinction between human–animal worlds seems softer. The Iñupiat live as though there is a normative truth expressed about how to treat the hunted animal contained in their metaphysical conception of the innate “powers” of animals (experienced phenomenally perhaps), which is transmitted between elders, hunters and the community as a whole. The transfer of moral knowledge takes the form of narrative, sometimes explicit and verbal, other times wordlessly conveyed. The enacted and expressed narratives highlight the relationship between the moral–spiritual and the practical. This truth also coincides with the very survival for members of the community. That is, this animal-integrated pragmatic view punishes those who are disrespectful towards the agency of animals lest dire consequences obtain. It is inaccurate to characterize the spirit-world as essentially diametrically opposite of the “real” world; in animistic worldviews (including among most Alaska Native traditions) the spirit and material worlds are 2 aspects of one existence, and are therefore unitary in a panenthistic way; so, these should be termed “material and spirit worlds” rather than “real and spirit worlds.” Further, many characterize the lack of animals not because they've remained in spirit form, but because in material form their spirit is aware of the hunter's lack of respect (as with most Dené groups).

8 These are categories often used to describe American pragmatism (18).
